# T-cell abnormalities in common variable immunodeficiency: the hidden defect

**DOI:** 10.1136/jclinpath-2015-203351

**Published:** 2016-05-06

**Authors:** Gabriel K Wong, Aarnoud P Huissoon

**Affiliations:** 1 MRC Centre for Immune Regulation, University of Birmingham, Birmingham, UK; 2 West Midlands Primary Immunodeficiency Centre, Birmingham Heartlands Hospital, Birmingham, UK

**Keywords:** IMMUNODEFICIENCY, AUTOIMMUNITY, CMV, IMMUNOGLOBULIN, LYMPHOCYTES

## Abstract

This review discusses how the T-cell compartment in common variable immunodeficiency is marked by the premature arrest in thymic output, leading to T-cell exhaustion and immune dysregulation. Although B cells have been the main focus of the disorder, ample experimental data suggest that T-cell abnormalities can be seen in a large proportion of Freiburg Group 1a patients and those suffering from inflammatory complications. The reductions in T-cell receptor excision circles, naïve T cells, invariant NKT cells and regulatory T cells suggest a diminished thymic output, while CD8 T cells are driven towards exhaustion either via an antigen-dependent or an antigen-independent manner. The theoretical risk of anti-T-cell therapies is discussed, highlighting the need for an international effort in generating longitudinal data in addition to better-defined underlying molecular characterisation.

Common variable immunodeficiency (CVID) is a heterogeneous and enigmatic primary immunodeficiency disorder marked by the failure of humoral immunity and immune dysregulation. The discoveries of T-cell-related molecular defects in hyper IgM syndrome and Inducible T-cell costimulatory (ICOS) deficiency highlighted the negative consequences to humoral immunity of the absence of T-cell support,^1^ ^2^ and growing experimental data are now suggesting that the T-cell compartment is also disrupted in patients with CVID.

CD4 T-cell lymphopenia was first noted among patients with CVID^3–5^ and can dramatically reverse the normal CD4:8 ratio when combined with expansion of senescent CD8 T cells, a feature not usually found in other antibody deficiencies.^6^ Despite the array of observed in vitro T-cell abnormalities, the clinical hallmarks of a T-cell immunodeficiency such as recurrent fungal and viral opportunistic infections are lacking in the majority of patients.

The heterogeneity of CVID and the progressive change in its definition over the years make comparing experimental data between studies difficult, but much can still be learnt. This article will examine existing experimental data in the literature regarding the role of T-cell defects in CVID and discuss how infection risk may increase progressively with decreasing thymic output, while the memory compartment shows evidence of abnormal activation. Additionally, we will assess these findings in the context of the revised 2014 European Society for Immunodeficiencies (ESID) diagnostic criteria and discuss their therapeutic implications.

## T-cells exhaustion

In addition to the quantitative alterations in CD4 and CD8 T cells, abnormalities in cytokine production and cell proliferation have been reported in CVID. Experimental data for cytokine output have been well summarised by Varzaneh *et al*,^7^ where sluggish production of interleukin (IL) 2 along with deficiencies in other cytokines such as IL-4, IL-5 and IL-10 were reported by a number of studies.^7–9^ On the contrary, high levels of inflammatory cytokines such as IL-6, interferon (IFN)-γ and tumour necrosis factor (TNF)-α were observed.^7^ During the acute phase of a viral infection in immunocompetent individuals, elevations in the classical trio of IL-2, IFNγ and TNFα are typically observed. By contrast, studies in chronic hepatitis C and HIV infections demonstrated that T-cell exhaustion, by phenotypic and functional alterations, following a long period of antigenic stimulation was typically marked by the sequential disappearance of IL-2 and then TNFα.^10^ ^11^ Therefore, the cytokine signature in CVID mimics an earlier phase of T-cell exhaustion.

Studies of T-cell proliferation also support the idea of a state of T-cell exhaustion. Earlier studies showed that T-cell proliferation to phytohaemagglutinin and OKT3 was normal in patients with CVID but responses may be suboptimal against tetanus toxoid or T-cell receptor (TCR) antibodies.^12^ Cyclic AMP-dependent protein kinase A type 1 (PKAI), an inhibitor of T-cell proliferation, can accumulate in exhausted T cells following prolonged antigenic stimulation.^13^ ^14^ Using a PKAI selective antagonist, Aukrust *et al*^15^ demonstrated that normal proliferation may be restored, suggesting that the poor T-cell proliferation in CVID was secondary to senescence.

Programmed cell death protein 1 (PD-1), an extended family member of CD28 and Cytotoxic T-lymphocyte-associated protein 4 (CTLA-4), functions as an important immune checkpoint for T cells and negatively regulates immune responses. In a murine lymphocytic choriomeningitis virus model, the synergistic use of IL-2 therapy and PD-1 blockade was able to reverse the exhausted immunophenotype of CD8 T cells and to regain control over the infection.^16^ Similar to the PKAI antagonist experience, Perreau *et al*^17^ showed that in vitro CD4 proliferation in patients with CVID may also be restored by blocking the programmed death ligand 1 and 2 axes (PD-L1/2). While anti-PD-1 antibodies have not been directly tested in CVID, clinical improvement was reported in patients who received IL-2 therapy in the past.^18^ ^19^ Along with suboptimal proliferation responses, a small number of studies also suggest an impaired apoptotic function contributing to the accumulation of effete T cells.^20–22^

Overall, both in vivo and in vitro data suggest the presence of potentially reversible T-cell exhaustion in some patients with CVID.

## Exaggerated CD8 T-cell responses

While the cause of T-cell exhaustion in CVID is not clear, comparable immunophenotypic features can be found with chronic viral reactivation, raising the possibility of an antigenic driver. T cells of patients with CVID, in particular CD8 T cells, exhibit high levels of activation and memory markers including CD29, CD38, CD95, CD45RO and Human leukocyte antigen (HLA)-DR, and low expression of CD27, CD62L and CD45RA.^23–26^ Although the expansion of T-cell memory is natural with advancing age, this process is greatly enhanced in CVID. Similarly, a disproportionate increase in terminally differentiated, senescent T cells (CCR7^−^CD45RA^+^) which are positive for CD57 and PD-1 was found across multiple studies, further supporting the presence of a persistent, unregulated cellular immune response in CVID.^6^ ^17^ ^26^ ^27^ Unlike T-cell proliferation and cytokine output, immunophenotypic findings are more consistent across studies and affect a larger proportion of patients with inflammatory complications such as polyclonal proliferation, chronic enteropathy, interstitial lung disease and autoimmunity.

To further support the presence of an underlying antigenic driver, CD8 T cells were shown to be oligoclonal by TCR spectratyping.^28–30^ As normal repertoire diversity can be seen in age-matched X-linked agammaglobulinaemia patients, this oligoclonality was not thought to be secondary to the deficiency in antibodies.^29^ In addition, some patients with CVID were shown to carry very dominant, stable CD8 clonotypes over time.^29^ Consistent with the notion of an underlying antigenic driver, a unique set of hyperexpanded TCRβ complementarity determining region 3 (CDR3) sequences could be found among patients with CVID. Although previous reports highlighted the preferential use of Vβ 4, 12 and 17 gene segments in these expanded clonotypes,^20^ ^31^ both Ramesh *et al*^32^ and our work could not reproduce these results using modern next-generation sequencing approaches and the level of oligoclonality was much more subtle.

Using a pentamer specific for HLA-A2 cytomegalovirus (CMV) peptide (NLV: *NLV*PMVATV), Marashi *et al* examined the role of CMV as a putative antigenic driver in CVID. A higher frequency (1–1.5%) of CMV NLV-specific CD8 T cells co-expressing high levels of IFNγ and TNFα was found in patients with CVID when compared with healthy controls (0.25%), while the frequency of Epstein–Barr virus-specific T cells was not increased.^33^ Similarly, Ki76 expression, a proliferation marker, in NLV-specific CD8 T cells was greatly increased in patients with CVID with inflammatory complications (2.4% vs 0.32%).^34^ Although causality could not be demonstrated and bystander activation of CMV-specific T cells could not be ruled out, an exaggerated CMV immune response appears to be closely associated with inflammatory complications in CVID.

Autoimmunity is another potential candidate driver for an exaggerated T-cell response. In patients with chronic diarrhoea, T-cell aggregates and nodular lymphoid hyperplasia are often found in intestinal biopsies, mimicking graft-versus-host disease.^35–38^ However, detailed examination of tissue T cells is technically challenging and it is currently not possible to differentiate if these histological features are driven by infections, for example norovirus,^39^ autoimmunity or other pathological mechanisms. Furthermore, a recent study suggested that tissue inflammation in CVID is largely driven by CD3^−^ innate lymphoid cells as opposed to T cells.^40^

Overall, chronic activation of CD8 T cells in CVID either via an antigen-dependent or antigen-independent manner is likely to contribute to T-cell exhaustion. While CMV appears to be a promising antigenic driver, the role of autoimmunity remains unconfirmed and awaits further study.

## Reduction in thymic output

Although current studies have not confirmed a putative antigenic driver, the exaggerated T-cell response could also be due to a lack of regulation. By peripheral blood immunophenotyping, Fevang *et al* were the first to demonstrate a lower frequency of CD4^+^CD25^+^FOXP3^+^ T cells, an immunophenotype considered characteristic of regulatory T cells (Treg), in patients with CVID. RNA transcript levels for FOXP3 in CD4 T cells were also lower in patients, particularly in those with splenomegaly. To further support this, the frequency of Treg was inversely proportional to neopterin, a serum inflammatory protein.^41^ Several studies had since confirmed the reduction in peripheral blood Tregs (CD4^+^CD25^+^FOXP3^+^ or CD4^+^CD25^+^CD127^−^) with the greatest deficiencies identified in patients with autoimmune cytopenias or Freiburg Group 1a.^42–45^ Carter *et al*^46^ also suggested an association between decreased Tregs and CD8 T-cell exhaustion in CVID.

Poor expressions of CTLA-4 and Glucocorticoid-induced TNFR-related protein (GITR) on Tregs were also noted, suggesting a functional deficit.^46^ ^47^ Tregs isolated from patients with CVID and autoimmunity had inferior suppressive function when cocultured with autologous CD4^+^CD25^−^ T cells, although it is not clear if this phenomenon is primary or secondary, such as thymic sequestration by chronic infections.^42^ CTLA-4 haploinsufficiency had been identified in cohorts of patients with CVID and impaired Treg functions.^48^ ^49^ An ongoing international collaboration is being carried out to estimate the prevalence of this mutation among patients with CVID.

Altogether, deficiency in Treg provides a logical explanation for the presence of overexpanded and exhausted CD8 T cells, as well as the development of autoimmunity CVID patients.

The examinations of other thymic derived T cells suggest that the reduction in Tregs may be part of a much broader picture. Invariant NK T cells (iNKT) are a subset of T cells that exhibit both characteristics of NK cells and T cells. They have highly restricted TCRs (Vα24/Vβ11) and are responsible for a range of immune responses, in particular the control of chronic viral infections.^50^ Using CD1d tetramers and Vα24/Vβ11 antibodies, Fulcher *et al*^51^ first demonstrated a significant reduction in iNKT among Freiberg Group 1a CVID patients. The numerical reduction of iNKT was later confirmed by other groups.^52^ ^53^ In one study, almost half of the patients had no circulating iNKTs. Stimulation with CD1d tetramer and α-galcer, a natural ligand for iNKT, also failed to adequately expand the iNKT population.^54^ The combined reduction in both Treg and iNKT highlighted a potential problem with thymic output, as both are considered as primary products of the thymus.^55–57^ In keeping with this, immunophenotypic data often reveal lower frequencies of naïve (CD45RA^+^CCR7^+^) CD4 and CD8 T cells in CVID. Other T-cell compartments such as central memory, effector memory and terminally differentiated T cells were relatively unaffected or proportionally increased in frequencies, suggesting that T-lymphopenia in CVID is predominantly restricted to the naïve pool in which a numerical deficit was confirmed by bead-calibrated absolute counting. In addition, a reduction in recent thymic emigrants (CD45RA^+^CD31^+^) was also shown.^6^ ^58^ Finally, the T-cell receptor excision circle (TREC) is a gene segment by-product of VDJ rearrangement not replicated during cell division and is considered as the gold standard in measuring thymic output and recent thymic emigrants.^59^ As much as a 10× fold reduction in TREC was reported in both CD4 and CD8 T cells of patients with CVID when compared with age-matched healthy controls.^60^ ^61^

Therefore, ample experimental data support the reduction in thymic output in CVID. As proposed by Liston *et al*,^62^ reduction in thymic output in other partial T-cell immunodeficiencies may promote autoimmunity by disrupting the balance between effector and regulatory T cells. It is, thus, not surprising that the majority of the findings described so far in this article gravitate towards Freiberg Group 1a patients who have a higher rate of autoimmune complications. The disappearance of iNKT would further hinder B-cell activation and memory formation.^63^ It is, however, important to note that many T-cell independent mechanisms such as CLEC16A and Transmembrane activator and calcium modulator and cyclophilin ligand interactor (TACI) polymorphism may also mediate autoimmunity,^64^ ^65^ and thymic failure thus represents just one of several mechanisms for patients with CVID to develop autoimmunity. In addition to the increased risk of infection and autoimmunity, T-cell lymphopenia would theoretically impair tumour surveillance, predisposing patients to both haematological and non-haematological malignancies as suggested by Brent and colleagues.^66^

## B-cell and T-cell collaboration

While poor thymic function could lead to the observed T-cell abnormalities in CVID, it offers little explanation for the humoral deficiency. With the exception of ICOS deficiency, the connexion between B-cell and T-cell abnormalities in CVID remains largely speculative.^2^ It is possible that the reduction in naïve T cells limits available cognate T cells to support humoral function. In keeping with that, the loss in naïve CD4 T cells was associated with increased infections.^20^ IL-21-producing follicular T-helper cells are derived from naïve T cells and play a crucial role in supporting germinal centre functions.^67^ However, conflicting evidence exists in the literature regarding their role in the disease process.^6^ ^68^

On the other hand, the combined failure of T cells and B cells warrants further examination of the common progenitors. Interestingly, bone marrow progenitor cells from patients with CVID were less able to form colonies when examined by the methylcellulose assay, indicating a problem even prior to thymic selection.^6^ ^58^ Consistent with this hypothesis, Serana *et al*^69^ showed that both TREC and Kappa-deleting element recombination circle (KREC) were proportionally reduced in CVID.^69^ Compared with severe combined immunodeficiencies such as deficiencies in JAK3, RAG1 and IL7RA, the reduction in TREC and KREC in CVID was only modest and partial.^70^ Similar to the excision circle analyses, telomere length of T cells and B cells correlated with each other, further supporting a common progenitor defect driving the simultaneous failure of B cells and T cells.^71^ However, telomere shortening was not consistently demonstrated in all studies and more data are required to support or refute this hypothesis.^72^

## Diagnostic and therapeutic consideration

Given the prevalence of T-cell abnormalities among patients with CVID, the recent redefinition of CVID by the ESID now emphasises on the use of T-cell immunophenotyping during the diagnostic workup.^73^ However, while there is a sound rationale to exclude patients with recurrent opportunistic infections and late-onset combined immunodeficiency,^74^ the arbitrary cut-offs for CD4 lymphopenia and naïve T-cell lymphopenia will exclude a proportion of patients (box 1). Patients with massive splenomegaly and pancytopenia are not particularly susceptible to opportunistic infections and may face diagnostic exclusion. As a significant amount of the T-cell data were generated before the introduction of the revised ESID criteria, it will be of interest to revisit T-cell functions under the new definition in the future.

Box 1 **ESID 2014 revised diagnostic criteria for common variable immunodeficiency^73^**

At least one of the following:

- Increased susceptibility to infection
- Autoimmune manifestations
- Granulomatous disease
- Unexplained polyclonal lymphoproliferation
- Affected family member with antibody deficiency

AND marked decrease of IgG and marked decrease of IgA with or without low IgM levels (measured at least twice; <2SD of the normal levels for their age)

AND at least one of the following:

- Poor antibody response to vaccines (and/or absent isohaemagglutinins);
- That is, absence of protective levels despite vaccination where defined
- Low switched memory B cells (<70% of age-related normal value)

AND secondary causes of hypogammaglobulinaemia have been excluded (see separate list below)

AND diagnosis is established after the 4th year of life (but symptoms may be present before)

**AND no evidence of profound T-cell deficiency, defined as 2 out of the following:**

- **CD4 numbers/microlitre: 2–6 years <300, 6–12 years <250, >12 years <200**
- **% naive CD4: 2–6 years <25%, 6–16 years <20%, >16 years 10%**
- **T-cell proliferation absent**

T-cell criteria are marked in bold.

AND no evidence of Ataxia telangiectasia (cafe-au lait spots, ataxia, telangiectasia, raised Alpha fetoprotein (AFP)).

ESID, European Society for Immunodeficiencies.

The success of checkpoint inhibitors such as anti-PD-1 antibodies used in some cancers and experimentally in T-cell exhaustion raises the question if they have a therapeutic role in CVID, with or without recombinant IL-2.^75^ However, the clinical application of such therapies is unexplored outside the oncology setting and untested in immunodeficient patients. In addition, there is a real risk of causing or exacerbating the autoimmunity that characterises the very patients who might benefit from such T-cell resuscitation.

Studies of T cells also highlight for the need for specific consideration for immunosuppressants and biologics in CVID. While corticosteroids and rituximab are now widely prescribed for cytopenias and inflammatory conditions in CVID,^76^ ^77^ our experiences with anti-T-cell agents are less well established with long-term follow-up data still lacking. Given the diminished thymic output, therapies targeting T cells should be very carefully considered and tailored or they risk compromising the existing T-cell immunity without replenishment from the thymus in patients with inflammatory complications or Freiburg Group 1a. In addition to improved molecular characterisation, future studies should focus on gathering longitudinal data in this area and test if latent viral control could be affected by immunosuppressive therapies.

Finally, the combined defect in T cells and B cells provokes a consideration for haematopoietic stem cell transplantation (HSCT) in CVID, the only cure for a combined immunodeficiency disorder. A recent multicentre retrospective study involving 25 patients of various clinical presentations, mostly for refractory immune dysregulation and lymphoma, showed that endogenous immunoglobulin production can be revived after a match-related donor or match-unrelated donor bone marrow transplant.^78^ However, the procedure carries a significant morbidity and mortality rate (48% overall survival), much exceed that of modern paediatric immunodeficiency HSCT (81–92%).^79^ Also, many patients still required immunoglobulin replacement afterwards, limiting its potential application.

## Conclusion

Orthogonal evidence from multiple studies indicates an arrest in thymic output coupled with exhaustion in the memory T-cell compartment in patients with CVID and inflammatory complications or Freiburg Group 1a. It is anticipated that the revised ESID diagnostic criteria will result in an increased focus on T-cell abnormalities in CVID and it will be of interest to re-examine many of the observed abnormalities in well-characterised cohorts under the revised criteria. Importantly, this review calls for additional care in the use of immunosuppressants in CVID, with close monitoring of outcomes, perhaps through an international database.

**Take home messages**

- Reduction in thymic output is a common feature of CVID.
- T cells are driven towards exhaustion either via an antigen-dependent or an antigen-independent manner.
- Additional care in the use of immunosuppressants is needed to preserve patients' existing immunity.
